# A comparison between reported therapy staffing levels and the department of health therapy staffing guidelines for stroke rehabilitation: a national survey

**DOI:** 10.1186/1472-6963-14-216

**Published:** 2014-05-14

**Authors:** Gabrielle McHugh, Ian D Swain

**Affiliations:** 1 Bournemouth University, Talbot Campus, Poole, Dorset, England; 2 Clinical Science and Engineering, Salisbury District Hospital, Salisbury NHS Foundation Trust, Salisbury, England

**Keywords:** Stroke, Stroke rehabilitation, Stroke therapy, Stroke recovery, Staffing resources for stroke

## Abstract

**Background:**

This study compared reported staffing levels for stroke care within UK in-patient stroke units to stroke strategy staffing guidelines published by the UK Department of Health and the Royal College of Physicians. The purpose was to explore the extent to which stroke teams are meeting recommended staffing levels.

**Method:**

The data analyzed in this report consisted of the detailed therapist staffing levels reported in the demographic section of our national survey to determine upper limb treatment in stroke units (the ATRAS survey). A contact list of stroke practitioners was therefore compiled primarily in collaboration with the 28 National Stroke Improvement Networks. Geographic representation of the network areas was obtained by applying the straight-forward systematic sampling method and the *N*^th^ name selection technique to each Network list. In total 192 surveys were emailed to stroke care providers around England. This included multiple contacts within stroke teams (e.g. a stroke consultant and a stroke co-coordinator) to increase awareness of the survey.

**Results:**

A total of 53 surveys were returned from stroke teams and represented 20 of the 28 network areas providing 71% national coverage. To compare reported staffing levels to suggested DoH guidelines, analysis was conducted on 19 of the 37 inpatient hospital care units that had no missing data for staff numbers, unit bed numbers, number of stroke patients treated per annum, average unit length-of-stay, and average unit occupancy rates. Only 42% of units analyzed reached the DoH guideline for physiotherapy and fewer than 16% of the units reached the guideline for speech & language therapy. By contrast, 84% of units surveyed reached the staffing guideline for occupational therapy. However, a post-hoc analysis highlights this as an irregularity in the DoH guidelines, revealing that all therapies are challenged to provide the recommended therapy time.

**Conclusions:**

Most in-patient stroke units are operating below the DoH guidelines and are therefore challenged in providing the recommended amount of therapy and patient time to facilitate optimal functional recovery for stroke patients.

## Background

Historically stroke was seen as an inevitable risk of aging and given a low priority within the National Health Service (NHS). The initiation of the Stroke Improvement Strategy in 2007 reprioritised stroke resulting in a continuous improvement process adopted by the Department of Health (DoH) to improve the effectiveness and efficiency of the delivery of a person-centred, stroke care service [1]. As a consequence, a major improvement has been made in the medical management of stroke and the delivery of acute stroke care services such that hospital stay has seen a significant reduction from a mean of 23.7 days in 2008 to a mean of 19.5 days in 2010. However, functional recovery continues to present a considerable challenge and discharge disability levels (using Barthel Index scores) remain unchanged since 2008 with 58% of patients having a functional impairment on discharge from hospital [2]. Moreover, the majority of stroke patients experience upper limb motor impairment and reduced ability to perform basic activities. Indeed, complete functional recovery of the upper limb was estimated to have occurred in only 5% to 34% of cases examined at six months post-stroke [3].

Consequently the National Sentinel Clinical Audit [2] highlighted concern about the small proportion of patients deemed appropriate by therapists for rehabilitative therapy while in hospital and the low proportion of these patients who then receive therapy. For example, only 33% of patients deemed appropriate for therapy by hospital therapists, received 45 minutes or more of physiotherapy per day during the weekday stay (i.e. Monday to Friday). In explanation, the National Sentinel Stroke Clinical Audit [2] surmised that therapist assessment of patient tolerance for treatment may be too low while offering no evidence of such. Moreover, the Sentinel Audit recommended a major review of therapy working practices – possible in view of the evidence suggesting that UK therapists are overburdened with administrative duties thus detracting from direct contact time with patients [4,5].

However, the various staffing guidelines proposed by the DoH, the RCP or the NHS are cited as ‘guidelines’ not evidence based. The 2012 National Clinical Guidelines for Stroke [6], for example, publishes guidelines for stroke unit staffing and for rehabilitation intensity by each of the relevant therapists that they state are ‘reasonable and achievable targets’ that were reached through debate and consensus of the intercollegiate stroke working party. Nevertheless staffing resources or workforce planning are pinned to these guidelines without clear understanding of how these guidelines were derived or how relevant and reliable they are. Therefore to explore whether the issue is more fundamental and systemic than therapist working practices or poor assessment of tolerance levels we make comparisons between various staffing guidelines and reported therapist staffing levels for stroke rehabilitation in NHS hospitals.

The data for this paper were extracted from the demographic section of our national ATRAS survey. This work was part of a larger Programme Grant for Applied Research (RP-PG-0707-10012) funded by the National Institute of Health Research (NIHR) that was tasked to investigate Assistive Technologies in the Rehabilitation of the Arm after Stroke (ATRAS). The ATRAS national survey was guided by a span-of-task Advisory Panel of 12 stroke specialists whose expertise ranged from stroke consultants and therapists to stroke coordinators. Ethical approval for the survey was obtained from Bournemouth University.

## Method

Under the direction of an Advisory Panel a focus group was attended by a range of stroke unit practitioners (e.g., stroke consultants and therapists) from the southwest region of England (*N* = 30) who scoped the content and design of the survey. The focus group highlighted some difficulties with data gathering - e.g. the diversity of care settings, the complexity of treating varying levels of impairment following stroke, and the time constraints on care providers to complete a survey. To address these issues, a two part survey design was adopted. Part A gathered demographic data about the care setting (e.g. acute or combined stroke unit); and the whole time equivalents (WTE) for all staff on the unit - physiotherapy (PT), occupational therapy (OT), speech and language therapy (SALT), nursing and medical staff. Rather than gather consensus on treatment options prescribed in a survey, Part B was designed as open-ended and free text to allow clinicians to describe the most common treatment interventions used in the unit to rehabilitate stroke patients’ upper limbs following stroke. The first iteration of the survey was pilot-tested among the Advisory Panel which resulted in minor word changes. Ten randomly selected stroke units completed the final iteration in advance of the national distribution to test the operational procedures. This data was entered in the database and suitable for final analysis. Notwithstanding that the overall aim of the survey was to delineate the extent of stroke rehabilitation provided across the whole of England during a patient’s first twelve months post-stroke, the secondary analysis presented here is based on the in-patient staffing levels extracted from the demographic section of the survey.

### Distribution

One key objective was for national distribution of the ATRAS survey. This was achieved by collaborating with the 28 Stroke Improvement Networks to compile a contact list of stroke clinicians. Stroke Improvement Networks are national NHS networks that connect stroke practitioners around England to co-ordinate and support the stroke care pathway which extends from in-patient care to community care. Contact lists of stroke clinicians in these geographic Network areas were obtained for 19 of the 28 Networks willing to co-operate with the research study and additional clinician contacts were obtained through the South West Stroke Forum and through the NHS Consultant’s Guide to cover the geographic areas from the 9 networks that did not provide any clinician contact information. Geographic representation of the network areas was done using the straight-forward systematic sampling method and the *N*^th^ name selection technique (using a uniform interval of every 13^th^ entry on the lists provided by each network) [7]. To cover the 28 network areas the survey was emailed to 192 stroke care providers. However, this included multiple contacts within each team (e.g. a stroke consultant and a stroke co-coordinator) to increase awareness of the survey. The original Dillman [8] approach was adopted to engage individuals with the project [9]. A minimum of 3 email prompts with non-responders was used and a minimum of 3 telephone follow-ups were made to participants if further clarification of their responses was needed.

### Participants

A total of 54 surveys completed by clinical teams were returned representing 20 of the 28 Network areas achieving 71% national coverage. One survey was unsuitable for further analysis as it described a research situation and therefore, was not typical clinical treatment or staffing. The 53 surveys represented stroke teams who worked across 77 settings – of which 37 identified as in-patient care (i.e. acute stroke units, combined stroke units, and stroke rehabilitation units) and 40 were post hospital care setting (e.g. Community Health Care, Outpatient Care). After undertaking several follow-ups we were unable to get data about annual patient numbers from two surveys. The remaining surveys reported a total of 16,632 patients treated annually for stroke – 13,954 in hospital setting (acute, combined and rehabilitation units) and 2,678 in post-hospital setting such as community health care.

### Data analysis

Surveys selected for this analysis were based on the following inclusion criteria: surveys completed by in-patient hospital care stroke teams; stroke teams self identifying as a discrete unit (e.g. acute stroke unit only); stroke teams with no missing data for staffing levels in their unit; and stroke teams provided full data per unit on average length of stay, number of beds and occupancy rates. However, and despite several follow-up phone calls to the stroke teams, some data fields remained incomplete. Therefore, we adopted the strategy of removing units with any missing data from further analysis rather than replacing the missing data with mean values which could lead to distortion when the intent of the analysis was to compare reported staffing levels to DoH national guidelines. Consequently, this paper reports the data from 19 of the 37 in-patient hospital stroke units.

Teams reported the WTE for all staff members (e.g. PT, OT, SALT, nurse and medical) in the unit and the proportion and number of stroke patients treated annually. To isolate staffing levels for stroke patients, we adjusted staffing WTE to reflect stroke specific WTE only. For example, if a team indicated that the stroke patients treated annually in the unit represented 80% of all patients in the unit, the staffing WTE was adjusted accordingly. Staffing levels were then converted to reflect staffing levels per 10 beds to be consistent with the reporting method in the DoH national guidelines. Comparisons were then made between the staffing WTE levels for PT, OT and SALT reported in the ATRAS survey and the DoH staffing ‘assumption’ and the DoH staffing ‘aspiration’ guidelines. The Stroke Strategy Staffing Assumptions grid published in the NHS Workforce Planning Resource [10], National Institute for Health and Clinical Excellence (Nice) Quality Standards Stroke Topic Expert Group Meeting [11] and the DoH’s Progress in Improving Stroke Care [12] provides the DoH staffing WTE assumption and an aspirational staffing WTE for stroke units. We also used the terms ‘assumption’ and ‘aspirational’ to be consistent with the labels from the Stroke Strategy Staffing Assumptions grid.

## Results

Extracts of the DoH Staffing Assumptions used from the grid are reproduced in Table 1. Table 1 also shows the average staffing numbers for PT, OT and SALT reported in the ATRAS survey for acute (ASU), combined (CSU), and rehabilitation (SRU) units respectively as well as the average staffing for the 19 units. The Table also reproduces the staffing levels reported in the 2006 National Sentinel Stroke Audit [13] and the actual staffing levels reported in the DoH report of 2010 [12].

**Table 1:** DoH staffing guidelines vs.reported stroke units staffing

**1a: DoH staffing guidelines per 10 beds**^**1**^				1b: Reported staffing WTE per 10 beds*			
	**PT**	**OT**	**SALT**		**PT**	**OT**	**SALT**
DoH staffing assumption	1.50	0.60	0.80	ASU (N = 5)	1.79	1.54	0.67
DoH staffing aspiration	3.70	3.30	1.40	CSU (N = 10)	1.43	1.20	0.53
DoH staffing levels^2^	1.30	1.10	0.40	SRU (N = 4)	0.89	0.86	0.43
Sentinel Audit^3^	1.30	1.00	0.30	Average (N = 19)*	1.36	1.18	0.50

^1^Source: NHS Workforce Planning Resource (2009) & Progress in Improving Stroke Care (National Audit Office, 2010).

^2^Median staffing levels reported in Progress in Improving Stroke Care (Department of Health, 2010).

^3^Median staffing levels reported in the 2006 National Sentinel Audit.

*Reported staffing numbers are average whole time equivalents per 10 beds for all in-patient units.

The data in Table 2 show the reported staffing numbers per unit for each of the therapies and the number of therapists per 10 beds calculated from the bed numbers provided per unit in the survey. We then projected the staffing numbers based on the DoH assumptions and aspirational levels to approximate the staffing numbers that should be in each unit.

**Table 2:** Reported staffing numbers and staffing per 10 beds compared to projected DoH guidelines

**Unit**	^**1**^**PT staff report**	^**2**^**PT per 10 beds**	^**3**^**DoH assum 1.5 PT per 10 beds**	^**4**^**DoH Asprt 3.7 PT per 10 beds**	^**1**^**OT staff report**	^**2**^**OT per 10 beds**	^**3**^**DoH assum 0.6 OT per 10 beds**	^**4**^**DoH Asprt 3.3 OT per 10 beds**	^**1**^**SALT staff report**	^**2**^**SALT per 10 beds**	^**3**^**DoH Assum 0.8 SALT per 10 beds**	^**4**^**DoH Asprt 1.4 SALT per 10 beds**
1	2.63	0.91	4.35	10.73	2.25	0.78*	1.74	9.57	1.28	0.44	2.32	4.06
2	2.55	0.80	4.80	11.84	3.14	0.98*	1.92	10.56	1.30	0.41	2.56	4.48
3	2.04	0.85	3.60	8.88	1.36	0.57	1.44	7.92	1.36	0.57	1.92	3.36
4	5.23	2.27*	3.45	8.51	5.70	2.48*	1.38	7.59	1.43	0.62	1.84	3.22
5	3.17	0.99	4.80	11.84	3.51	1.10*	1.92	10.56	2.11	0.66	2.56	4.48
6	4.83	1.61*	4.50	11.10	3.85	1.28*	1.80	9.90	1.00	0.33	2.40	4.20
7	4.41	1.84*	3.60	8.88	2.84	1.18*	1.44	7.92	1.47	0.61	1.92	3.36
8	0.77	0.26	4.50	11.10	0.48	0.16	1.80	9.90	0.29	0.10	2.40	4.20
9	1.96	1.40	2.10	5.18	1.47	1.05*	0.84	4.62	0.49	0.35	1.12	1.96
10	4.00	1.00	6.00	14.80	3.00	0.75*	2.40	13.20	0.80	0.20	3.20	5.60
11	5.50	1.96*	4.20	10.36	5.00	1.79*	1.68	9.24	2.50	0.89*	2.24	3.92
12	3.60	1.50*	3.60	8.88	2.25	0.94*	1.44	7.92	1.80	0.75	1.92	3.36
13	3.92	1.23	4.80	11.84	2.02	1.26*	1.92	10.56	1.76	0.55	2.56	4.48
14	3.60	1.44	3.75	9.25	2.40	0.96*	1.50	8.25	0.40	0.16	2.00	3.50
15	7.50	2.21*	5.10	12.58	8.10	2.38*	2.04	11.22	3.10	0.91*	2.72	4.76
16	4.80	1.71*	4.20	10.36	4.00	1.43*	1.68	9.24	2.00	0.71	2.24	3.92
17	2.20	1.22	2.70	6.66	1.50	0.83*	1.08	5.94	1.00	0.56	1.44	2.52
18	1.80	0.60	4.50	11.10	1.50	0.50	1.80	9.90	0.60	0.20	2.40	4.20
19	6.00	2.00*	4.50	11.10	6.00	2.00*	1.80	9.90	3.00	1.00*	2.40	4.20

^1^Reported staff per unit.

^2^Reported staff per unit converted to staff per 10 beds.

^3^Staff required to reach DoH Staffing Assumption.

^4^Staff required to reach DoH Staffing Aspiration.

*Number of units reaching DoH staffing assumption for therapy per 10 beds.

**Figure 1:**
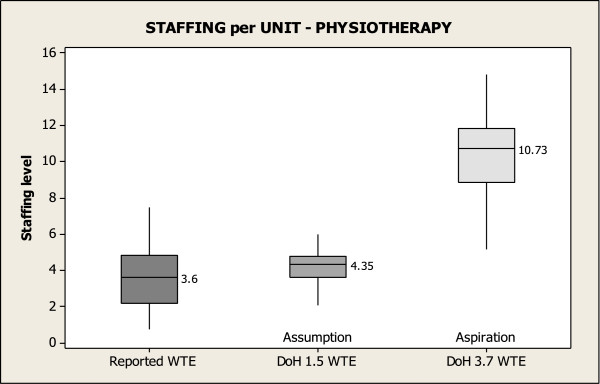
Physiotherapy staffing per unit.

**Figure 2:**
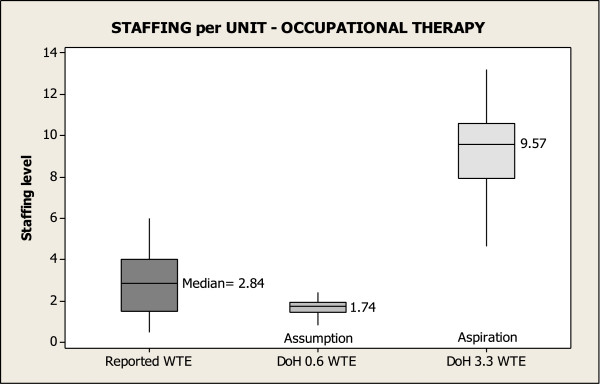
Occupational therapy staffing per unit.

**Figure 3:**
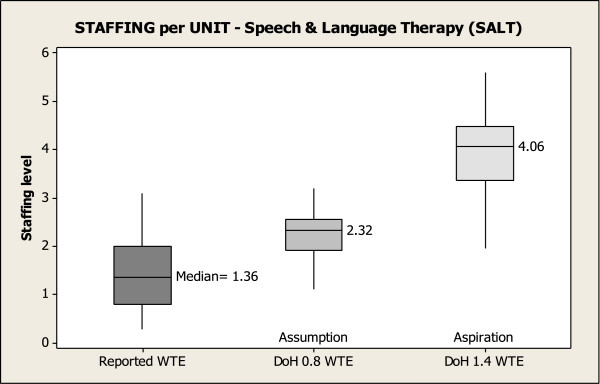
Speech & language therapy staffing per unit.

Figures 1, 2 and 3 show reported staffing levels per unit for PT, OT and SALT per 10 beds compared to the DoH staffing assumption and the DoH staffing aspiration for each therapy per 10 beds. Each Figure reads as follows; the first box depicts the reported number of therapists for the 19 units, showing the median staffing WTE per 10 beds for each of the therapies. Box 2 compares therapist staffing levels that ought to be in each unit, based on the number of beds reported in the survey and the DoH staffing assumption. Lastly, box 3 compares staffing levels based on the reported number of beds and the DoH staffing aspiration. These figures reveal the disparity between the reported staffing levels in units and the DoH staffing assumption and the DoH staffing aspiration. Only 42% of units reach the DoH assumption for PTs per 10 beds, and fewer than 16% of units reach the guide for SALT. None of the units surveyed were near the aspirational levels of staffing.

Figure 2 indicates that the reported OT staffing per 10 beds exceeded the DoH staffing assumption of 0.60 per 10 beds in 16 of the 19 units surveyed. This was a finding that contrasted with the low staffing levels for PT and SALT. However, we were unable to discover the origins or rationale for the low OT staffing levels proposed in the DoH national guidelines. Therefore we conducted a post hoc analysis to further explore the irregularity in the DoH national guidelines that would propose such a low staffing assumption for OT at 0.6 WTE per 10 beds compared to the WTE for SALT and PT (0.80 and 1.5 respectively). Consequently, our post hoc analysis was based upon:

(a) the percentage of patients deemed suitable for each of the three therapies proposed in the 2011 National Sentinel Clinical Stroke Audit [2] as 74% for PT, 69% for OT and 50% for SALT. Using these percentages adjusts for patients who would not receive therapy, for example, those patients who may be receiving end of life care.

and (b) the percentage of staff time spent in direct patient contact for PT, [5] OT [5], and SALT [14] extracted from previous studies at 46%, 33% and 25% respectively.

Our values were calculated in order to provide the recommended 45 minutes of each therapy to suitable patients per day over a 37.5 hour working week. Therefore, given 450 minutes per day (7.5 hour work day), one therapist could provide the recommended amount of treatment to each of 10 patients if the therapist only treated patients. Using the percentage of patients suitable for treatment and percentage of direct contact time per therapy this gives the formula – 10 beds divided by percentage of direct patient time multiplied by percentage of patients deemed suitable for therapy. Table 3 presents the staff requirements as 1.7 PT, 2.1 OT and 2.0 SALT per 10 beds in order to provide the recommended 45 minutes of each therapy per day. This staffing level reflects the higher percentage of direct patient time for physiotherapists (46% compared to 33% and 25% for OT and SALT respectively) resulting in a lower staff ratio than that of OT or SALT.

**Table 3:** Required staffing levels per 10 beds

	**PT**	**OT**	**SALT**
Percentage of patients suitable for treatment^1^	74%	69%	50%
Percentage of direct contact time^2^	46%	33%	25%
Resulting number of therapists required per 10 beds	1.6	2.1	2.0

^1^Percentage of patients appropriate for treatment extracted from the National Sentinel Stroke Clinical Audit (2010, p39).

^2^Percentage of direct contact time reported by Putman, et al. [14] and Pring et al. [15].

## Discussion

The 2010 National Sentinel Stroke Clinical Audit [2] raised concern about the small proportion of patients deemed appropriate for rehabilitative therapy by therapists and the low level of therapy actually received by these patients during their hospital stay. The report concludes that a major review of therapist working practices was warranted. However, no convincing evidence was proposed to support a need for a major review of working practices in the first instance. Consequently, the purpose of this paper was to further explore the issue, but from a more fundamental rationale of staffing levels, rather than a higher-order rationale of staff practices or assessments by comparing the reported staffing levels in the ATRAS survey to DoH national guidelines [10-12].

What is evident from the data is that few units surveyed met the DoH guidelines for PT and SALT – only 42% and 16% of units reached the guideline for PT and SALT respectively. The low staffing level reported in the ATRAS survey is consistent with the National Sentinel Stroke Audit [13] which, for example, recorded a median of 0.3 SALT per 10 beds (range 0.2-0.6) in 2006 compared to the DoH staffing assumptions of 0.8 SALT per 10 beds. In the current ATRAS survey only 3 of the 19 units had staffing levels that reached the DOH guidelines indicating the staffing for SALT still remain unacceptably low. Additionally, the DoH Survey of Stroke Unit Staffing and Patient Dependency [15] similarly reported in 2007 that only 25% of units had adequate staff numbers for rehabilitation.

It is necessary to reiterate here that the guidelines proposed by the various bodies are approximations of staffing levels deemed ‘reasonable and achievable’ [6] to deliver stroke care. However, and despite our search, we have found no rationale for the original staffing assumption or aspirational levels to inform us how these numbers were established or the method by which they were derived. We can offer no insight into the relevance, reliability or reasonableness of these numbers upon which the complexities of stroke care rest. We draw attention to the paucity of information because of the oddity from the data when we analyzed the staffing levels for occupational therapy.

When we based our analysis on the DoH staffing assumptions – notwithstanding that these assumptions are even lower than the actual staffing levels reported in the 2006 National Sentinel Stroke Audit [13] and the actual staffing levels reported in the DoH report of 2010 [12] - Figure 2 suggests 84% of units have staffing levels for occupational therapy that reach or exceed the DoH staffing assumptions. However, our post hoc analysis points to the irregularity of this finding. In this analysis it was calculated that 2.1 OTs (adjusting for percentage of patients deemed appropriate for treatment and percentage of direct patient time) are required per 10 beds in order to provide the recommended amount of therapy (i.e. 45 minutes per day, 5 days a week). By contrast, the DoH staffing assumption is only 0.6 OTs per 10 beds – lower than the DoH assumption for SALT. Again, as we were unable to ascertain exactly how the DoH figures were derived it is difficult to comment on where errors might have arisen. We recognise the simplicity of our calculation; nevertheless, what is obvious is that our post hoc analysis gave figures for PT of 1.7 per 10 beds comparable to the DoH staffing assumptions of 1.5 per 10 beds. By contrast, the OT staffing levels were strikingly different between our calculations giving 2.1 per 10 beds compared to only 0.6 in the DoH staffing assumptions. The DoH figure is difficult to explain given that in their aspirational levels the DoH give comparable levels of PT and OT quoting 3.7 and 3.3 per 10 beds respectively.

Consistent with reports that outcomes for stroke patients appear worse for UK patients than the rest of Europe [16], DeWit et al. [4] reported that UK patients were significantly less likely to be in therapy than patients in Germany, Belgium or Switzerland and found that more than 35% of UK therapy time consisted of nursing care compared to 5% in Switzerland and Germany and 10% in Belgium. Furthermore UK patients spent under 12% of their time interacting with occupational therapists compared to 29% in Switzerland, 25% in Germany, and 20% in Belgium. The staffing levels reported here further confirm that stroke units are challenged in providing the recommended therapy time proposed in the RCP Clinical Guidelines for Stroke, [6]. Furthermore, the 2010 National Sentinel Clinical Stroke Audit [2] found that only half of all patients with motor deficits were deemed appropriate for 45 minutes of therapy on 2 or fewer weekdays within the first 28 days of stroke. The National Sentinel Clinical Stroke Audit pointed to therapist working practices in explanation for the low level of therapy provided. By contrast, the findings here suggest staffing levels are actually well below those needed to enable the provision of recommended therapy.

Despite the evidence to suggest that intensive rehabilitation improves functional recovery outcomes for patients [17,18] the findings from the Langhorne et al. study [19,20] found early involvement of physiotherapy in patient care in 67-100% of units and the early involvement of occupational therapy and speech and language therapy in only 34-66% of units. Not surprisingly the NICE guideline for stroke [8] encourages greater usage of active treatment that provides the opportunity for repetitive practice of movement for patients. However, this cannot be achieved without appropriate staffing levels. The basic premise in this paper is that the majority of stroke care units have staffing issues that pose a challenge to the provision of the recommended rehabilitation to enable optimal functional recovery for stroke patients. What can be inferred from our numbers is that the prioritisation of stroke revolves around medical management and has yet to extend into optimal functional recovery. Further analysis of our data, extrapolating patient-to-therapist ratios, is provided elsewhere [21] and further supports our premise that the provision of patient-centred rehabilitative care remains a challenge despite the significant improvement in the medical management of stroke.

One of the limitations is that our analysis was based on complete data provided by only 19 in-patient hospital care units, although these units did account for over 13,000 patients. However, we made the decision not to enhance our numbers by using mean values to fill empty cells so our data would be an authentic reflection of unit level analysis. Nevertheless, the research does reveal staffing limitations in providing therapy for stroke patients. Most stroke units are operating below the DoH staffing assumption levels and are therefore challenged in providing the recommended amount of therapy and patient time to facilitate optimal functional recovery for stroke patients. Additionally selection bias could be considered a limitation in that we randomly sampled within a well targeted population of stroke practitioners who had a strong interest in stroke improvement as demonstrated by their involvement in the Stroke Improvement Networks. However our strategy was deliberate. The survey requested very detailed information about each stroke unit and to maximize participant engagement we sought out the stroke units we believed would be more likely to collaborate with our study. Therefore it may be that we have surveyed the ‘crème-de-la-crème’ of stroke units and the data should be interpreted accordingly.

Another limitation is that we did not clearly differentiate between therapists and therapy assistants in our calculations. Although our numbers are based on therapist grades ranging from band 3 to band 8a, the majority of the reported therapists were within bands 5 to 8a. Indeed bands 5 to 8a represent the therapists who have the skills and competencies to deliver specialized stroke care such as constraint induced movement therapy or electrical stimulation. Even the RCP clinical guidelines [6] recognize the evidence in favour of specialized stroke units therefore it is reasonable to expect these specialists will be at upper rather than lower bands. To argue for the inclusion or exclusion of lower bands from our calculations may be redundant as Turton and Pomeroy [18] still determined that patients are suffering from too little practice to optimize recovery.

## Conclusions

This survey has clearly demonstrated that:

• current staffing levels pose a challenge to the provision of rehabilitation enabling optimal functional recovery for stroke patients.

• only 42% of units studied reached the DoH staffing assumptions for physiotherapy.

• fewer than 16% of the units reached the DoH staffing assumptions for speech and language therapy.

• that 84% reach the staffing assumption for occupational therapy reflects an irregularity in the DoH staffing assumption guide.

• providing the recommended 45 minutes of each therapy per day to patients who are deemed appropriate for treatment [2] requires a staffing level estimated at 1.7 PT, 2.1 OT and 2.0 SALT per 10 beds reflecting the adjustments we made for direct contact time – 46%, 33% and 25% respectively [5,14]. By this calculation fewer PTs are required per 10 beds as they have the greatest percentage (46%) of direct time with patients. This results in a staffing configuration quite different from clinical practice but logically consistent – PTs have higher direct patient contact than OTs and SALTs.

In conclusion, medical management of stroke has demonstrated significant improvement (as seen in the reduction of patient hospital stay) in the delivery of stroke care documented in consecutive sentinel audits. Nevertheless, discharge disability scores are not demonstrating similar improvements in the equivalent time span. In recognition of and with due respect to the experts who have had the difficult task of proposing guidelines, our hope with this paper will engender debate and future research on how to optimize a patient’s functional recovery post stroke.

## Abbreviations

ASU: Acute stroke unit; ATRAS: Assistive technologies in the rehabilitation of the arm after stroke; CSU: Combined stroke unit; DoH: Department of health; LOS: Length of stay; NHS: National health service; Nice: National institute for health and clinical excellence; NIHR: National institute of health research; OT: Occupational therapist; PT: Physiotherapist; SALT: Speech and language therapist; SRU: Stroke rehabilitation unit; WTE: Whole time equivalent.

## Competing interests

The authors declare that they have no competing interests.

## Authors’ contributions

GMcH is responsible for the data collection and data analysis, as well as for reporting the study results. IS was the Chief Investigator and NIHR grant applicant and has read and approved the final manuscript. Both authors read and approved the final manuscript.

## Pre-publication history

The pre-publication history for this paper can be accessed here:

http://www.biomedcentral.com/1472-6963/14/216/prepub
